# Experimental evidence for scale-induced category convergence across populations

**DOI:** 10.1038/s41467-020-20037-y

**Published:** 2021-01-12

**Authors:** Douglas Guilbeault, Andrea Baronchelli, Damon Centola

**Affiliations:** 1 The Haas School of Business, The University of California, Berkeley, Berkeley, CA 94720 USA; 2 Network Dynamics Group, The University of Pennsylvania, Philadelphia, PA 19106 USA; 3 Department of Mathematics, City University London, London, EC1V 0HB UK; 4 The Alan Turing Institute, London, NW12DB UK; 5 Annenberg School for Communication, The University of Pennsylvania, Philadelphia, PA 19106 USA; 6 School of Engineering, The University of Pennsylvania, Philadelphia, PA 19106 USA; 7 Department of Sociology, The University of Pennsylvania, Philadelphia, PA 19106 USA

**Keywords:** Evolution of language, Sociology

## Abstract

Individuals vary widely in how they categorize novel and ambiguous phenomena. This individual variation has led influential theories in cognitive and social science to suggest that communication in large social groups introduces path dependence in category formation, which is expected to lead separate populations toward divergent cultural trajectories. Yet, anthropological data indicates that large, independent societies consistently arrive at highly similar category systems across a range of topics. How is it possible for diverse populations, consisting of individuals with significant variation in how they categorize the world, to independently construct similar category systems? Here, we investigate this puzzle experimentally by creating an online “Grouping Game” in which we observe how people in small and large populations collaboratively construct category systems for a continuum of ambiguous stimuli. We find that solitary individuals and small groups produce highly divergent category systems; however, across independent trials with unique participants, large populations consistently converge on highly similar category systems. A formal model of critical mass dynamics in social networks accurately predicts this process of scale-induced category convergence. Our findings show how large communication networks can filter lexical diversity among individuals to produce replicable society-level patterns, yielding unexpected implications for cultural evolution.

## Abstract (web summary)

Category systems exhibit striking agreement across many cultures, yet paradoxically individuals exhibit large variation in the categorization of novel stimuli. Here the authors show that critical mass dynamics explain the convergence of independent populations on shared category systems.

## Introduction

People exhibit substantial creativity and variation in how they categorize novel and ambiguous phenomena^1–6^. This observation has led decades of research to argue that category formation in large social groups is unpredictable^7–13^. Larger populations contain a greater diversity of people and thus a greater diversity of categories that can be adopted through communication networks, which are expected to lead to variable and path-dependent cultural trajectories^8–10,14–18^. Meanwhile, there is considerable evidence that independent populations consistently arrive at highly similar category systems across a range of topics^19–21^, including flora^22^, geometry^23^, emotion^24^, color^25^, and kinship^26^. These findings pose a striking puzzle—how is it possible for separate and diverse populations, composed of individuals with significant variation in how they categorize the world, to independently construct similar category systems^19,27–29^?

One explanation for the observed patterns of category convergence across societies is that there are innate universals in human psychology that arise independently of social interaction^10,19–22,30^. However, because these theories explain similarity across populations in terms of innate human categories, they are limited in explaining how category convergence can emerge when individuals widely vary in their categorization of novel stimuli^1–6,10^. An alternative view holds that stochastic dynamics can lead separate large populations to arrive at similar category systems even when individuals vary in how they categorize the world. Formal models of voting behavior, for instance, show that increasing sample size can increase the likelihood of identifying the most popular choice in a population for both binary^31^ and pluralistic choices^32,33^. Similarly, recent findings on critical mass dynamics^28,34^ suggest that large populations have the potential to promote the interpersonal spread of popular linguistic conventions. Building on this work, our formal analyses indicate that when the popularity of categories can be described by a hypergeometric distribution (or binomial for infinite populations), then increasing population size can trigger “scale-induced” category convergence, in which a small number of categories are more likely to consistently reach critical mass^34^ and spread^35,36^ in large populations, resulting in replicable evolutionary trajectories (see Supplementary Information sections 1.1, 1.2, and 1.3 for model specification).

An empirical test of these predictions has not yet been possible because it requires comparing the cultural trajectories of independently evolving small and large populations to observe whether differences in population size directly affect the similarity of the category systems that populations produce. In this study, we developed an online experimental platform called the “Grouping Game” that enabled real-time observation of novel category formation in small and large populations (see ‘Methods’). We use the Grouping Game to investigate this puzzle experimentally by examining how small and large populations independently construct category systems for a continuum of novel and ambiguous stimuli. Solitary individuals and small groups produced highly divergent category systems. Yet, across replicated studies with unique subjects, separate large populations converged on highly similar category systems. These findings offer insight into category similarities across societies^19–22^, by showing how large communication networks can filter lexical diversity in such a way that leads communities toward convergent and replicable trajectories in category creation.

## Results

**Fig. 1:**
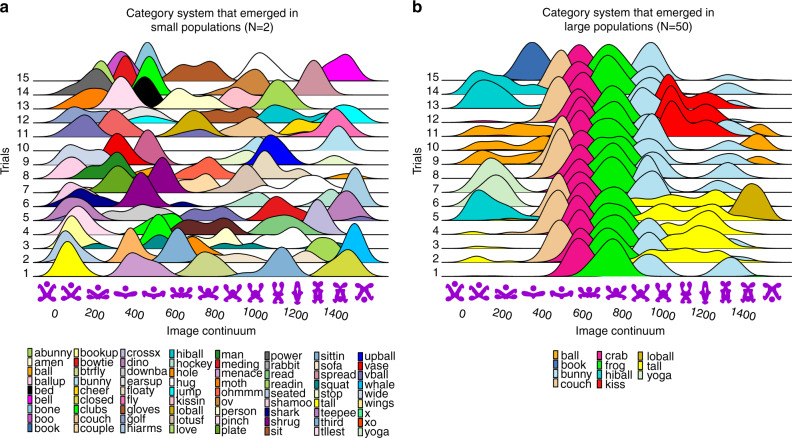
Larger populations promote category convergence across populations. Comparing the level of convergence in category systems that emerged in small (*N* = 2) (**a**) and large (*N* = 50) (**b**) populations. Each row displays the category system constructed by a single unique population in each condition after 100 rounds of interaction. The horizontal axis displays the image continuum of shapes, consisting of 1500 slices. Density distributions display the frequency of successful coordination for each label, as well as the region of the continuum to which each label referred. Each color indicates a unique label. Similarity in the category systems across independent populations indicates convergence.

Figure 1 displays the category systems that emerged in distinct small and large populations. Figure 1a shows that small populations (*N* = 2) produced highly divergent category systems. Only 6% of labels were shared across independent dyads, and there was no consistency in how these dyads partitioned the continuum (*p* < 0.001, *n* = 80, Kruskal–Wallis *H* Test). As a result, dyads varied not only with respect to the labels they adopted for the same regions of the continuum, but also with respect to the regions of the continuum they successfully categorized. (Complementary analyses showing the same results for the *N* = 1 condition are provided in the Supplementary Information section 1.7; Figs. S6 and S7). By contrast, large populations (*N* = 50) generated remarkably similar vocabularies (50% Jaccard Index, *p* < 0.001, *n* = 95, Wilcoxon Rank Sum Test, two-sided) and similar partitions of the continuum (*p* = 0.87, *n* = 15, Kruskal–Wallis *H* Test), indicating convergence in how these independent populations categorized the novel stimuli (Fig. 1b).

These findings appear puzzling at first since larger populations are expected to increase the unpredictability of category formation as a result of containing a greater diversity of individuals, and thus a greater number of categories that are introduced and available for adoption. Yet, our results indicate that increasing population size—and thereby increasing the diversity of categories—can counterintuitively lead to convergent trajectories in category formation across populations.

**Fig. 2:**
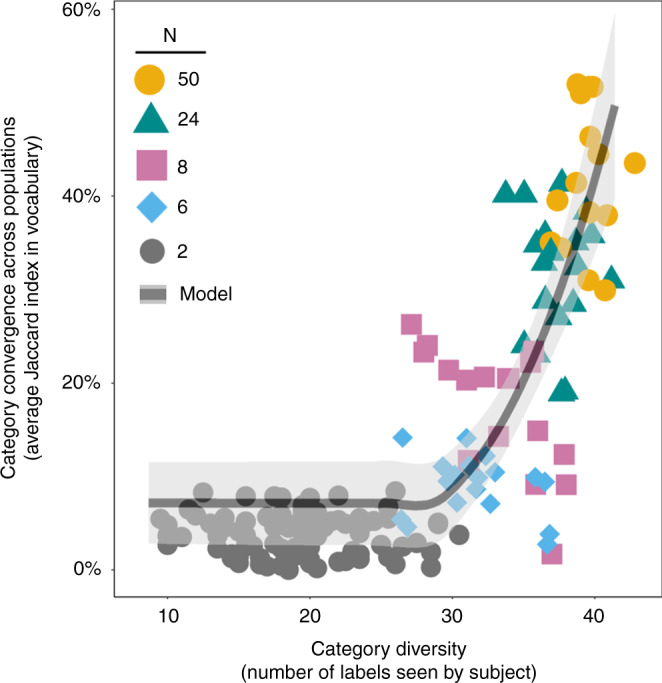
Convergence in the vocabularies that emerged in populations of different sizes, for *N* = 2 (black dots), *N* = 6 (blue diamonds), *N* = 8 (purple squares), *N* = 24 (green triangles), and *N* = 50 (yellow circles). Vertical axis reports the average similarity in vocabulary (average Jaccard Index) between each network trial and all other networks of the same population size. Horizontal axis displays category diversity, measured as the average number of unique labels encountered by subjects in a population. Data points represent experimental results (80 dyads and 15 social networks of each size). Black trend line shows model predictions (averaged over 50 simulated trials; 100 rounds each trial; *d*_min_ = 0.01; |*L*| = 5000; *b* = 1); see Supplementary Information section 1.2 for model specification (equation S1). The measure of center indicated by the model trend line is the mean Jaccard Index among simulated trials of the same population size, ordered by the average category diversity in each trial (Fig. S1). Error bands show 95% confidence intervals.

Our theoretical predictions for these convergence dynamics provide an excellent fit with our experimental findings (Fig. 2) (see Supplementary Information section 1.2 for model specification; Fig. S2). Across all experimental conditions, label diversity significantly increased with population size (*p* < 0.001, *n* = 120, Jonckheere-Terpstra Test). Figure 2 shows that greater label diversity within populations predicts greater similarity in the category systems that emerge between populations of the same size (*p* < 0.001, *n* = 120, Jonckheere-Terpstra Test). We find these convergence dynamics not just for the labels that were used, but also for how participants partitioned the continuum into distinct regions (Supplementary Information section 1.8; Fig. S8).

Robustness experiments (Supplementary Information section 1.9) show that providing more rounds of interaction for the dyads (>125) did not increase their rate of convergence. Instead, it further entrenched their divergent category systems.

**Fig. 3:**
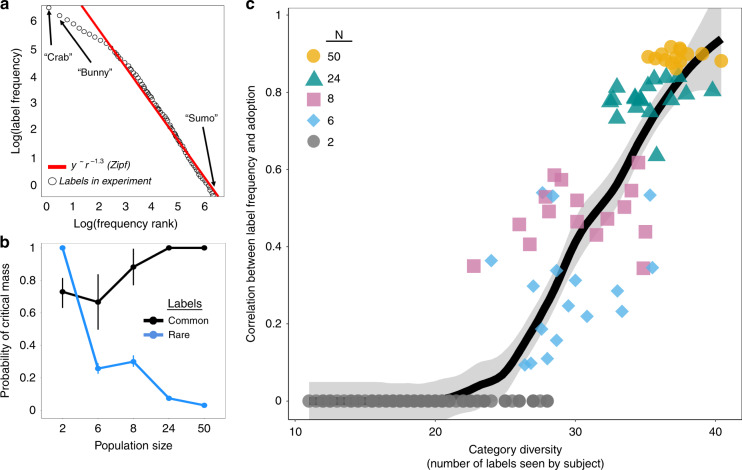
Larger populations amplify the spread of initially frequent labels. **a** Using the Zipf distribution to model the initial frequency of labels (including data from all conditions; *N* = 2, *N* = 6, *N* = 8, *N* = 24, and *N* = 50), where initial frequency refers to the number of individuals who introduced a label without any prior exposure to the label in the task. Vertical axis displays the log of each label’s initial frequency. Horizontal axis displays the log of each label’s frequency rank. **b** Displaying the mean effect of population size on the ability for labels to reach critical mass (when at least 25% of subjects in a network independently introduce a label). Common labels are identified as outliers with high initial frequency (Supplementary Information section 1.3). Data display the proportion of experimental trials in each condition for which each label type reached critical mass. Error bars display 95% confidence intervals. **c** The correlation between the initial frequency of a label in a population and the proportion of subjects in a population who adopted the label (vertical axis), where adopting a label entails that a subject produced a label after being exposed to it. Horizontal axis displays the diversity of categories in each trial, indicated as the average number of unique labels encountered by each subject in a network. Error bands display 95% confidence intervals. All observations are independent and at the network-level. All panels represent data from 80 unique dyads and 15 unique social networks of each size.

We propose a simple mechanism to explain our findings. We suggest that larger populations amplify the spread of initially more frequent labels^37^, leading these common labels to reach a “tipping point”^34^, after which they diffuse and become widely adopted^35,36^. Figure 3a shows the frequency with which every label was independently suggested by participants across all studies. Consistent with Zipf’s law^38^, a small number of labels like “crab” and “bunny” were common, meaning they were more likely to arise separately from distinct participants, whereas the vast majority of labels were rare, meaning they were only introduced by a small number of individuals (Fig. 3a).

Figure 3b shows the relationship between population size and critical mass dynamics (formal model and detailed analyses provided in the Supplementary Information section 1.3; Fig. S2). In small populations, common labels were not sufficiently reinforced to reach the tipping point needed to trigger widespread adoption^35,36^. Consequently, small populations (*N* = 2) were significantly more likely to adopt rare labels (*p* < 0.001, *n* = 80, Wilcoxon Signed Rank Test, two-sided), leading these populations to follow divergent evolutionary trajectories. However, increasing population size significantly increased the likelihood that common labels (like “crab” and “bunny”) would be reinforced and adopted (*p* < 0.001, *n* = 120, Jonckheere-Terpstra Test), while significantly reducing the likelihood that rare labels would spread (*p* < 0.001, *n* = 120, Jonckheere-Terpstra Test). Our findings indicate a direct relationship between population size and category convergence across independent populations (Fig. 3c). For large populations (*N* = 50), the likelihood of common labels becoming widely adopted approaches unity, leading to consistent and replicable trajectories in collective category formation (Fig. 3b, c and S2).

A crucial implication of our theory is that category similarities across social groups do not solely depend upon cognitively salient features of the labels themselves, but also upon the labels’ frequency in the population. An established intuition is that certain categories gain popularity because they have intrinsic appeal (e.g., because of their ‘natural’ descriptive fit with the stimuli)^39^. However, even when the most popular labels (e.g., “crab” and “bunny”) were attempted in dyads, they regularly failed to gain acceptance (Supplementary Information section 1.3; Fig. S2). This suggests that the adoption of these labels is not strictly determined by their cognitive appeal, but rather by the fact that they are more likely to be reinforced and reach critical mass in larger populations.

**Fig. 4:**
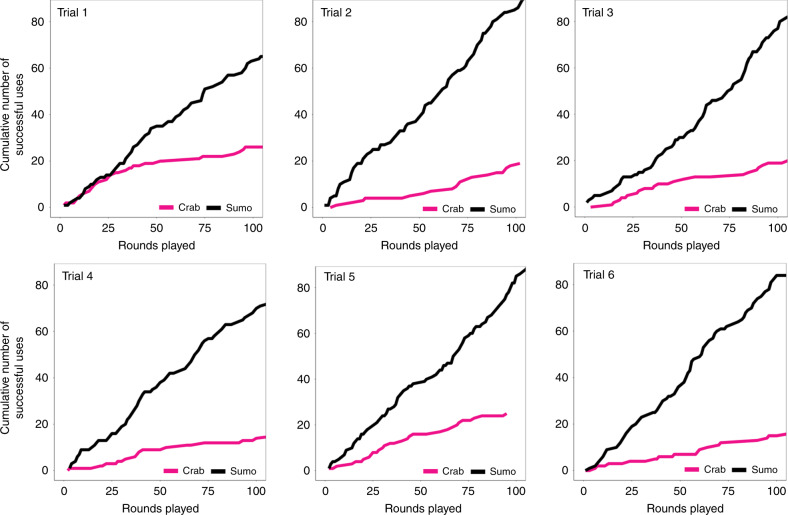
Time series showing the adoption of the confederates’ rare label (“sumo”) by experimental subjects (i.e., nonconfederate subjects). Pink lines indicate the cumulative number of successful uses among experimental subjects of the label “crab”. Black lines indicate the cumulative number of successful uses among experimental subjects of the label “sumo”. Each round is measured as *N*/2 pairwise interactions, such that each player has one interaction per round. The data displayed exclude all interactions between confederates.

To evaluate this hypothesis, we experimentally tested the following counterfactual: if we artificially inflated the popularity of infrequent labels to reach critical mass, would this trigger convergence on those labels rather than on more cognitively appealing ones? We conducted six robustness trials (*N* = 24) in which each network contained a minority of confederate subjects (37%) tasked with spreading a novel category system based on infrequent labels (see Supplementary Information section 1.10 for full details on experimental design; Fig. S9). For instance, we trained confederates to use the rare label “sumo” (Fig. 3a) for the same regions of the visual continuum associated with the most popular label in our initial studies, “crab” (Fig. 1b). Figure 4 shows that although “crab” appeared in each robustness trial, “sumo” consistently outcompeted “crab”. In every robustness trial, populations adopted the confederates’ labels across each region of the continuum, yielding significantly more convergent category systems (58% Jaccard Index) than those that emerged in *N* = 24 populations without confederates (35% Jaccard Index) (*p* < 0.01, *n* = 21, Wilcoxon Rank Sum Test, two-sided; Figs. S10–S12).

## Discussion

The “social constructivist” view of cultural evolution suggests that large communication networks contain greater individual variation, which leads to greater divergence and unpredictability in the evolution of category systems^7–15,18^. Here, we show that while increasing the size of communication networks does, in fact, significantly increase the diversity of categories that people encounter, it does not increase divergence. Rather, it increases category convergence across independent populations. Our results suggest that convergence in category formation across independent populations is significantly shaped by the communication networks in which people are embedded.

These findings offer experimental insight into past observational data on category similarities across societies^19–26^. Our findings suggest that communication in large social networks can help filter cognitive and lexical diversity in such a way that promotes the replicable development of similar category systems across separate communities. Importantly, we observe scale-induced category convergence for an arbitrary and novel continuum of stimuli that lacks pre-existing objective boundaries, whereas some mathematical models assume that well-defined objective boundaries are essential for producing stable convergence dynamics in the emergence of vocabularies^40–42^. We anticipate that future research may extend our findings to study how population dynamics can improve both the stability and accuracy of category systems in domains with objective truth conditions. In particular, we anticipate that future studies may apply our findings to address challenging issues in content moderation and classification, for instance to eliminate individual biases in large-scale citizen science efforts and related human crowdsourcing tasks, such as Galaxy Zoo^43^ or Gravity Spy^44^, and to improve consistency in the classification of acceptable and unacceptable content on social media^45^.

## Methods

This research was approved by the Institutional Review Board at the University of Pennsylvania, where the study was conducted, and it included informed consent by all participants.

**Fig. 5:**
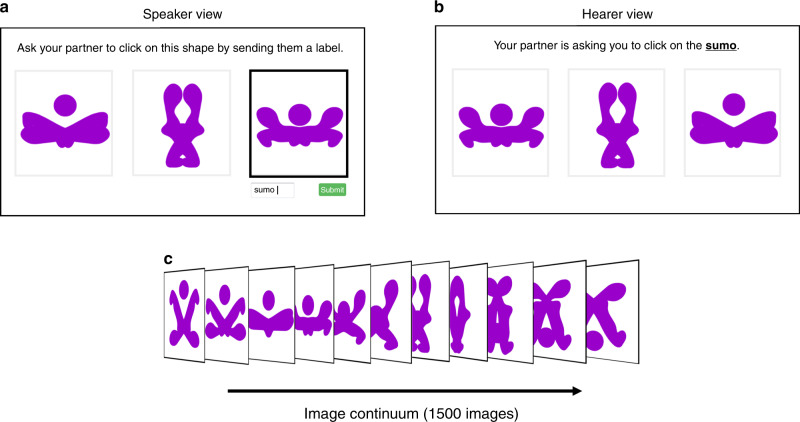
The Grouping Game. Screenshots of “The Grouping Game” interface from the view of a speaker (**a**) and the view of a hearer (**b**) on a given round. **c** A sample of the continuum of novel shapes used as stimuli in the experiment.

A total of 1480 subjects were recruited from Amazon Mechanical Turk to participate in an online language game^28,34,46^ called “The Grouping Game” (Fig. 5). Each trial consisted of unique individuals, producing independent experimental observations. All subjects were required to live in the U.S. with English as their first language. When logging into the Grouping Game, subjects were randomized into either a dyad, or a network of 6, 8, 24, or 50 people. We conducted additional trials using an alternative version of the Grouping Game constructed for solitary players (*N* = 1), which generated results consistent with our findings for dyads (Figs. S2 and S3). We collected 80 dyads (*N* = 2) and 15 social networks for each population size (i.e., *N* = 6, *N* = 8, *N* = 24, and *N* = 50). There were no differences in the distribution of demographic traits across conditions, in terms of gender (*p* = 0.56), ethnicity (*p* = 0.42), and age (*p* = 0.67) (Kruskal–Wallis *H* Test). All data were collected between September 2018 and February 2020.

We created a continuum of novel shapes that defined the space of visual stimuli for the Grouping Game (Fig. 5c). Analogous to the visible color spectrum, our continuum was a smooth geometrical progression that was not inherently partitioned^27^. We evenly divided this continuum into 1500 slices. Each slice was a unique shape.

Upon arriving to the study, participants viewed instructions on how to play the Grouping Game. In the game, participants played a series of pairwise one-shot coordination games, where a single coordination game constituted a single round. In each round, participants were randomly paired with another participant in their network. In all conditions, participants could be paired with any other participant, creating fully connected (i.e., homogeneously mixing) populations.

Each round of the game proceeded as follows. First, each subject was randomly paired with another subject in their network (in the dyads, participants were always paired with the same person). Second, in each pair on each round, one subject was randomly assigned to be the “speaker” (Fig. 5a) and the other was the “hearer” (Fig. 5b). Third, the speaker in each pair was shown three randomly selected slices (or shapes) from the visual continuum, which were presented side by side (Fig. 5). One of the three shapes was randomly highlighted only for the speaker. The speaker was given 30 seconds to enter a label of their own creation into a free text-entry window, with the aim of helping their partner to distinguish the highlighted shape from the other two presented shapes. The only restriction on label production was that speakers were not allowed more than six characters to prevent highly detailed sentence-like descriptions that could not fail to coordinate. Even with this character limit, nearly 5000 unique labels were introduced. Fourth, the hearer in each pair was shown the same set of three shapes as the speaker but in an alternate order (Fig. 5b). The hearer was then given 30 seconds to identify the shape corresponding to the speaker’s label (Fig. 5b). If the hearer selected the correct shape, both players received a successful payment (10¢). If the speaker failed to select the correct shape, both players were financially penalized (1¢). Every experimental trial of the Grouping Game lasted 60 min. In every trial, each subject played at least 100 rounds.

The image continuum was held constant across conditions. In every trial, every subject was presented with a uniform distribution of images drawn equally from all regions of the continuum (see Supplementary Information section 1.4; Fig. S3). The algorithm that randomly selected three images to display each round was designed so that participants were never shown the same shape twice. All images displayed for a given scene were at least 75 frames apart along the continuum, following prior theoretical models^27,47^. This design induced subjects to categorize the images, because in this environment, subjects would only use the same label on multiple rounds if they were grouping distinct images under a single category. The set of three images displayed on each round were unique to each pairing, such that two separate speaker and hearer pairs interacting at the same time would see distinct image sets on a given round.

Participants had no information about the labels used by other members of the population except for their partner’s response in the round in which they were paired^28,34^. In every network (*N* = 2, *N* = 6, *N* = 8, *N* = 24, and *N* = 50), subjects received identical instructions. Subjects did not have information about their partner’s identity, nor the size of their network. A manipulation check confirms that subjects’ knowledge about their network was held constant across experimental conditions (Supplementary Information section 1.5; Fig. S4). Any differences in the category systems that emerged across experimental conditions can be attributed to the direct effects of population size on the dynamics of category formation.

To identify the categories that emerged in each condition of each trial, we used DBSCAN (Density-based spatial clustering of applications with noise)^48^. A key advantage of DBSCAN is that it does not require one to specify the number of clusters in the data a priori, as opposed to *k*-means clustering. The DBSCAN algorithm involves two key parameters: *MinPts*, which determines the minimum number of points that must be included in each cluster, and *ε*, which denotes the radius of the neighborhood around a point *x* that is used when identifying clusters. For each condition in each trial, we ran DBSCAN to identify clusters of labels based on their values along two features: the total number of successful coordination events associated with a label across the entire population, and the total number of cumulative adopters overtime associated with each label. We ran DBSCAN separately for each condition in each trial because increasing population size significantly increases the number of possible successful coordination events and adopters that can be associated with a label. Following standard methodology, for each application of DBSCAN^48^, *MinPts* was set to 3 (the number of dimensions plus one) and *ε* was chosen by plotting the *k*-distances among points and using the knee of the plot to identify the optimal *ε*. Emergent categories were identified as the unique cluster of labels with the highest values in terms of their total number of successful uses and their total number of adopters. In practice, DBSCAN identified 3–5 labels as emergent categories. All results are robust to varying vocabulary size across a wide range of fixed sizes (see Supplementary Information Section 1.6; Fig. S5). In cases where two categories were successful for the same region of the continuum, the label with the highest number of coordination successes was deemed the most successful category for this region.

### Reporting summary

Further information on research design is available in the Nature Research Reporting Summary linked to this article.

## Supplementary information

Supplementary Information

Reporting Summary

Supplementary Software

## Data Availability

The data underlying this study are publicly available at: https://github.com/drguilbe/categories2020; https://ndg.asc.upenn.edu/uncategorized/network-dynamics-of-category-emergence/. Source data are provided with this paper.

## Source data

Source Data

## Code availability

The source code for this study is publicly available at: https://github.com/drguilbe/categories2020; https://ndg.asc.upenn.edu/uncategorized/network-dynamics-of-category-emergence/.
