# Supplementary material for: Experimental evidence for scale-induced category convergence across populations
Source: Nat Commun. 2021 Jan 12;12:327. doi: 10.1038/s41467-020-20037-y (PMC7804416; doi:10.1038/s41467-020-20037-y)
Supplement: Supplementary file 1 — Supplementary Information [file 41467_2020_20037_MOESM1_ESM.pdf]

Supplementary Information for

# Experimental Evidence for Scale-Induced Category Convergence across Populations

**This file includes:**

Supplementary Analyses

Supplementary References

## 1. Supplementary Analyses

### 1.1. The Categories Model

We reproduce our experimental results in a formal model that extends prior formal models of the "Categories Game" (*1,2*). The Categories Game recreates a paradigmatic scenario in language interaction – famously codified by Wittgenstein (*3*) – where a speaker uses a word to direct the actions of a hearer. The categories model involves a population of $N$ individuals (or players), committed in the categorization of a single continuous perceptual channel, where each stimulus is represented as a real-valued number ranging in the interval [0, 1). The range [0,1) creates a fully continuous novel dimension. The model defines categorization as a partition of the interval [0, 1) in discrete subintervals, denoted as "perceptual categories".

For the negotiation dynamics in the model, each individual agent is initialized with a dynamic inventory of form-meaning associations linking their perceptual categories (the partitions of the continuum) to labels, where the "meaning" of the label is how it maps onto a subset of perceptual categories in the continuum. Perceptual categories and the labels associated with them co-evolve dynamically through a sequence of elementary communication interactions ("language games"), referred to as rounds within a game. All players are created with only the trivial perceptual category [0,1], with no label associated to it. At each time step, a pair of individuals (one playing as speaker and the other as hearer) is selected and presented with a new "scene": i.e., a set of $M \geq 2$ objects (stimuli), where $O_i \in [0, 1)$ with $i \in [1, M]$.

One of the objects in $M$ is highlighted as the *topic*, and the speaker's task is to draw the hearer's attention to the *topic* by communicating a label. The speaker starts by discriminating the scene, if necessary, adding new category boundaries to isolate the topic; then she labels one object and the hearer tries to guess it. The label to name the object is chosen by the speaker among those associated to the category containing the object, with a preference for the one that has been successfully used in the most recent game involving that category. In a successful game, both players erase competing words in the category containing the topic, keeping only the word used in that game; in failed games, the speaker points out the topic and the hearer proceeds to discriminate it, if necessary, and then adds the spoken label to its inventory for that category (*1,2*).

The model also accounts for the fact that agents are limited in their ability to distinguish objects/stimuli that are too close to each other in the perceptual space. In a given scene, the $M$ stimuli are chosen to be at a distance larger than this threshold: i.e., $|o_{i} - o_j| > d_{min}$. Prior work demonstrates that the results do not qualitatively vary by $d_{min}$. Prior work also shows that altering the size of $M$ does not alter the qualitative outcomes of the model, but only the amount of time it takes for convergence (*1,2,4*).

### 1.2. Extending the Categories Model

A limiting assumption in the original Categories Model is that the labels proposed by agents are arbitrary, since they are defined as random strings. As a result, each new label proposed by an agent in the model bares no prior relation to the simulated continuum or to the labels already in

use in the population. When agents introduce new labels, it is not possible for some labels to be introduced at greater frequency than others. Thus, while separate populations in the model consistently converge on a finite vocabulary, there is no process to guide their convergence dynamics toward similar labels with similar partitions of the continuum. The result is path dependence independent of population size – i.e. variation in the category systems across separate populations (*1*,*2*,*4*).

We extend the prior model of the categories game by including population biases in label production. We add a global set of possible labels *L* defined as a sequence of real values on the range [0, |*L*|] from which all agents in a network draw when introducing new labels. We define a probability distribution over *L*, denoted as $L_{B,}$ where $_B$ refers to population bias. We use the Zipf distribution, which holds that the size of the $r^{th}$ largest occurrence of the event (in this case, the frequency of a word) is inversely proportional to its rank: $y \sim r^{-b}$, with $-b$ close to unity. The Zipf distribution is chosen not only because it has been widely established as characteristic of word frequencies in a population, but it also closely describes the distribution of label frequencies introduced in the experimental data reported in the main text (Fig. 4).

The value of *b* can be altered to adjust the steepness of the probability distribution. Higher values of population bias increase the likelihood that a small set of possible labels will be introduced independently by separate agents. At extremely high values of *b*, all agents will independently select the same label. Decreasing *b* thereby increases the amount of variation in the population.

By defining *L*, we test the hypothesis that (i) in the case of no bias in label production, population size will have no effect on the similarity of category systems that emerge in separate populations, and (ii) that in the case with minimal population bias, increasing population size will amplify the spread and adoption of the same labels across separate populations. We measure cross-group similarities in category systems in terms of the average pairwise Jaccard index between the vocabularies that emerged in separate populations of the same size. All networks are initialized as fully-connected networks.

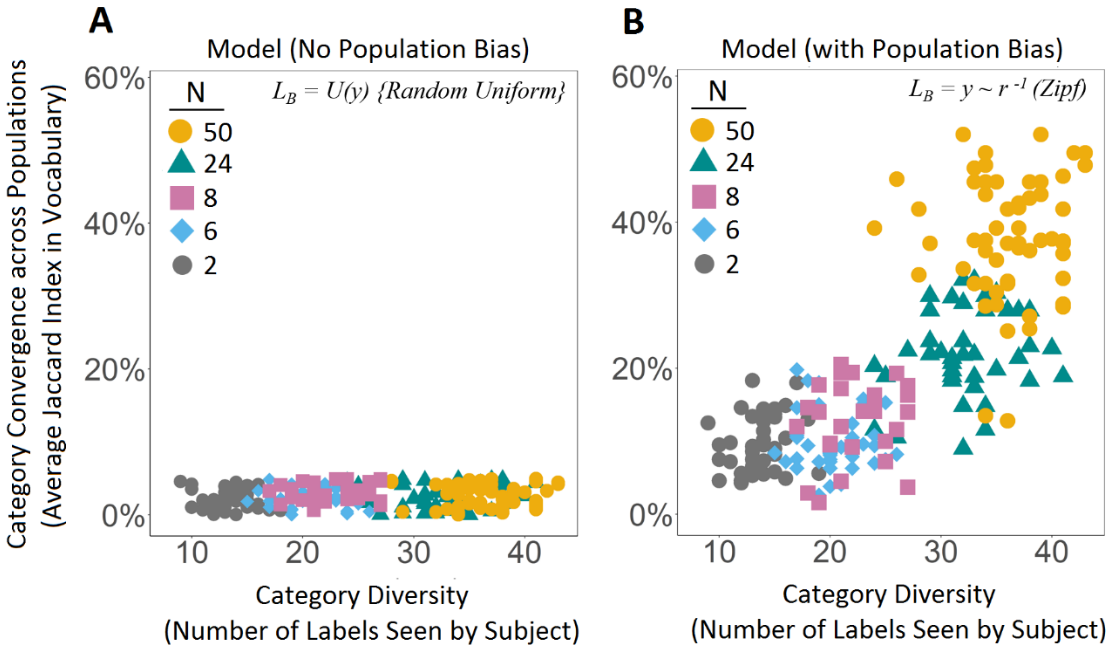


Fig. S1. Results of 50 simulations (100 rounds; $d_{min} = 0.01$; $|L| = 5000$) comparing the effects of population size on bias and category convergence across populations. Each data point represents a single network in each condition, where the horizontal axis indicates the level of category diversity in a population (i.e. the average number of unique labels that agents encountered in that population). (A) Convergence in vocabulary after 100 rounds in networks of varying population size where the model is initialized with $L_B$ (the level of population sampling bias) is random and uniform, indicating no bias. (B) Convergence in vocabulary after 100 rounds in networks of varying population sizes where the model is initialized with $L_B$ defined by the standard Zipfian distribution (i.e. where $y \sim r^{-b}$, and $b$ approximates unity).

We compare the effect of population size on vocabulary convergence across populations under varying conditions of population bias, while defining $|L| = 5000$ (which is approximately the number of unique labels observed in the experiment). The modeling results provide strong support for the hypothesis that when $L$ is defined with no population bias (Fig. S1*A*), there is no convergence above chance in any network size. By contrast, the model shows that when $L$ is defined by minimal population bias using the standard Zipfian ($y \sim r^{-b}$), with $b \approx 1$, increasing population size significantly increases the amount of vocabulary similarity observed between separate networks of the same population size (Fig. S1*B*).

1.3. <u>A Mathematical Model of the Effect of Population Size on Critical Mass Dynamics for Label Diffusion</u>

The question of whether a particular label is likely to reach a critical mass within a population of size $n$ is analogous to the infamous birthday paradox (*5*): the counterintuitive result in probability

theory where the extremely unlikely event that two randomly sampled individuals share the same birthday becomes vastly more likely as the size of the sample population increases. Once a population reaches at least 23 people, it becomes more likely than chance for two people in this population to share a birthday (*5*). Once populations surpass 50 people (the size of the largest population in our experiment), it becomes almost mathematically guaranteed that two people in the population will share a birthday (*5*).

By analogy, the problem of whether a label reaches critical mass within a population is a question of the probability that a certain proportion of people in the population introduce the same label (i.e., "have the same birthday"). But the problem of critical mass involves two subtle differences that require formalization. First, the question of whether a label reaches critical mass in a population is not about the *specific* proportion that introduces the label, but about whether the proportion of the population introducing the label reaches the *minimum requirement* to trigger a tipping point. Secondly, the distribution of label frequencies is not uniformly distributed: some labels are more likely to be introduced than others (Fig. 4*A*). Recent work shows that the birthday paradox holds with nonuniform distributions, though its formalization rarely incorporates this subtlety (*6*). Both of these additional features of critical mass can be readily modeled using the hypergeometric distribution (and technically the binomial distribution for infinite populations).

The hypergeometric distribution is derived by computing the probability of selecting $k$ successes in a sample of size $n$ from a total population of size $N$, where the total frequency of successes in the population is given by $K$. To determine the likelihood of obtaining *at least* $k$ successes in a sample of size $n$, we sum the probabilities of obtaining $k$, $k + 1$, …. $k + (n – k)$ successes using the following formula.

$$Probability\ of\ at\ least\ k\ successes\ for\ all\ k\ in\ [k, \dots, n] = \sum_k^n \frac{\binom{K}{k}\binom{N-K}{n-k}}{\binom{N}{n}} \quad (1)$$

A "success", for our purposes, refers to selecting an individual from the population who introduces label $x$. The total number of possible successes in $N$ (i.e. $K$) corresponds to the number of individuals who will introduce label $x$. Within this model, we can distinguish rare from common labels by altering the probability of an individual introducing $x$ at baseline; in other words, by modifying $K$ as a function of the probability $P(x)$ of an individual introducing label $x$. For instance, in our experiment, 38% of people on average introduced the label 'crab' within our test population of 1480 subjects. Therefore, assuming $K = N \cdot P(x)$, we arrive at $K$ = 562. By contrast, a rare label like 'sumo' was introduced by 0.07% of the population, so $K$ = 10.

The final component of this model incorporates the critical mass size of interest, denoted by *cm*. The theory of critical mass dynamics concerning the spread of a single convention (*7*) shows that when $cm \approx 25\%$, then a committed minority can gain sufficient levels of social influence to trigger a global shift in adoption of the convention; however, *cm* can vary according to key parameters like memory length and resistance (*7*). In the observed convergence dynamics

of large populations ($N$=50) in our experiment, the average $cm$ was 20%. To incorporate this element into equation (1), we constrain the model so that for each $n$, we set $k$ as the min($k$) such that $k/n \geq cm$.

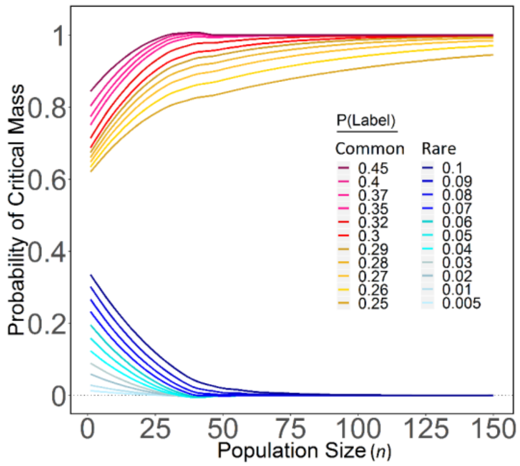


Fig. S2: Using the hypergeometric distribution to model the effect of population size on the likelihood of labels reaching critical mass. Horizontal axis displays the size of a population sample ($n$) from the total population size $N$, which is set to 1460 to emulate the size of the test population in our main experiment. The vertical axis displays the probability of a label reaching critical mass (20%). The colors indicate the probability of an individual being drawn who uses a given label. Rare labels are highlighted by cooler colors, and common labels are highlighted by warmer colors.

Drawing from analytic properties of the hypergeometric distribution, we show that population size directly affects the likelihood of common labels reaching critical mass. Fig. S2 displays these results while assuming $cm$ = 20%, following prior research (*7*). We find that common labels with $0.25 \leq P(x) \leq 0.45$ are more likely to reach critical mass in larger populations ($n$>20), where the probability they will reach critical mass approaches unity when $n$>50. These results indicate that common labels are much more likely to reach critical mass and spread in larger populations, as a result of the properties of the hypergeometric distribution. These results are robust to a range of $cm$ values.

These findings provide a clear response to the following objection: what if we observe higher levels of category convergence across larger populations because large populations have a higher chance of containing people who produce cognitively 'better' labels that will always succeed when introduced? (*8*) This would imply that whenever these 'better' labels appeared in the dyads, they should have succeeded. Surprisingly, we find that even in small populations when common labels were introduced, rare labels were regularly adopted in their place. As an example, we located the region of the continuum shared among all uses of the common label

"crab" in the $N$=50 networks (i.e. images in the range 500 – 600), and we examined the dyads in which "crab" was attempted for this region. Every time this label was introduced for this region in large networks ($N$=50), it gained adoption. By contrast, even in dyads where the label "crab" was attempted for this same region, a wide range of rare labels were adopted in the place of "crab", including "baby", "turtle", "hotdog", and "smile". As a result, dyads were much less likely to adopt "crab" when this label was introduced compared to $N$=50 populations ($p < .001$, CI = [0.63, 0.83], Binomial Test).

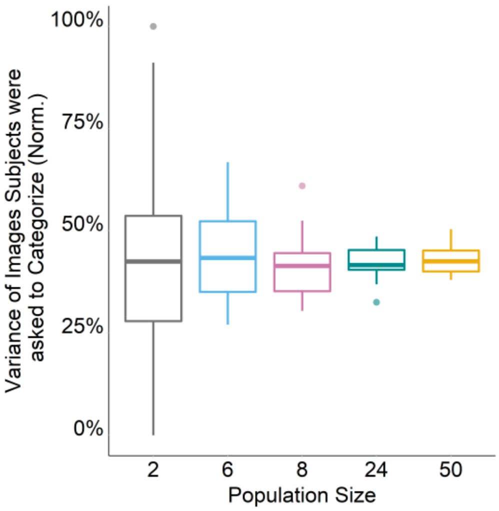


Fig. S3: Comparing conditions in terms of the distribution of shapes that subjects were asked to categorize. Boxplots display the variance of the shapes that each individual was asked to categorize, averaged first at the trial-level. Data points indicate outlier trials. The data displayed represent 80 unique dyads and 15 unique social networks of each size. Data are represented using min-max normalization. Norm., normalized.

1.4. <u>Robustness to Variation in Image Selection</u>

Here we test whether the design of our image selection algorithm contributes to differences in patterns of category convergence across conditions. To address this concern, Fig. S3 shows that the variance of the shapes that individuals were asked to categorize did not significantly vary by condition. Regardless of condition, individuals saw nearly the same spread of shapes from across the continuum. This is also indicated by the fact that there were no significant differences in the raw distribution of images that individuals saw across conditions ($p$ = 0.48, Kruskal-Wallis H Test). As well, we verify that our results are robust to the rare case of repeated images. While the algorithm was design to never show subjects the same shape twice, there were rare cases when (i) the algorithm could not find a set of images that neither the speaker nor hearer had seen before, or (ii) the available images satisfying (i) violated a distance constraint of 75 images (which was imposed following prior models; *1,2,4*). We confirm that in the rare cases when

subjects were shown the same image twice in social networks, no image was more likely repeat, preventing algorithmic bias in the process of social reinforcement ($p$ = 0.59, Kruskal-Wallis H Test). All results hold when excluding these repeat images.

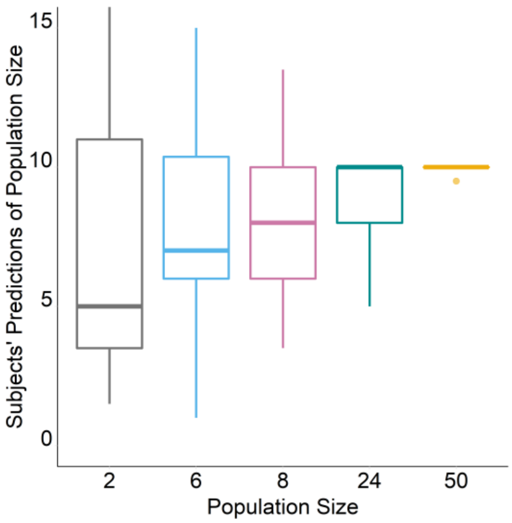


Fig. S4. Subjects' beliefs about the size of the population they were playing with during the game. Subjects' provided their estimates in an exit survey after the interactive phase of the game was complete. Horizontal axis displays the experimental condition based on population size. Boxplots display the mean and distribution of subjects' population size predictions in each condition, which is first computed by taking the median prediction for each trial in each condition to control for nonindependence. Data points indicate outlier trials. The data displayed represent 80 unique dyads and 15 unique social networks of each size.

1.5. Confirmation of Manipulation Check Regarding Subjects' Perceptions of Group Size

Regardless of experimental condition, subjects were not given any information about the size of the group they were in, and subjects received identical instructions both before, during, and after the game. All instructions were standardized to ensure that group size served as a precise control variable for our experimental manipulation, such that group size was the only difference between the design of each condition. As a manipulation check, subjects were presented with a survey immediately after the experiment in which they were asked to report the number of other players they thought they were playing with during the game. We find a surprising amount of variation within each population size, such that subjects were generally inaccurate in their perceptions of group size in all conditions (on average, participants in all group sizes estimated their groups to consist of somewhere between 5 and 10 people) (Fig. S4). There were no statistically significant differences in the predicted population size among experimental conditions ($p$ = 0.92, F=0.22).

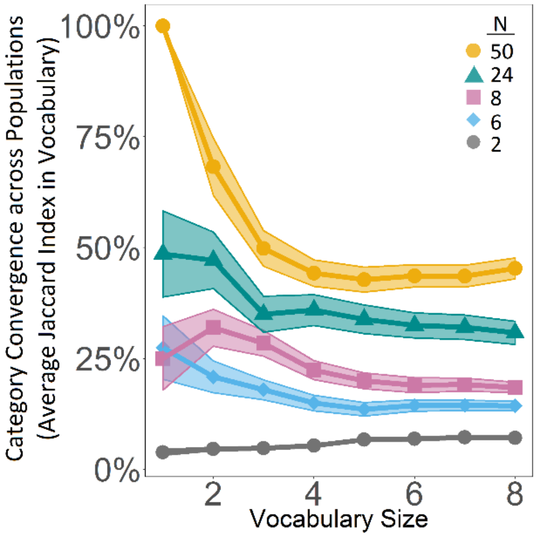


Fig. S5: Measuring convergence in vocabularies across experimental populations while holding vocabulary size constant. The horizontal axis displays the size of the vocabulary imposed on each trial in each condition when computing the average Jaccard index between the vocabularies of all trials within each condition. The measure of center displayed along the vertical axis shows the mean Jaccard index across all populations of the same size. Error bands display 95% confidence intervals.

1.6. Robustness to Vocabulary Size

Our main results are based on the use of DBSCAN to identify the categories that emerged within each condition in each trial. This approach permits the size of the vocabularies of different social groups to vary. However, the DBSCAN algorithm requires one to manually specify two parameters (*MinPts* and $\varepsilon$), and changes to each of these parameters can significantly affect the number of clusters the algorithm identifies. Here we show that all of our main results are robust to holding vocabulary size constant and comparing the cross-group similarity of category systems across the full range of reasonable vocabulary sizes. For each condition in each trial, vocabulary size *n* is determined by calculating the top *n* labels with the highest cumulative number of successful uses across the population. Fig. S5 displays the cross-group similarity of the category systems that emerged for each vocabulary size. We find that larger social groups produce higher levels of cross-group category systems across all reasonable vocabulary sizes.

1.7. Robustness to Category Formation among Isolated Individuals

We also designed a separate web application that allowed subjects to categorize the continuum on their own, without any social interaction (Fig. S6). In the individual version of the task, participants were shown a subset of 50 equally spaced images from the continuum. All individuals viewed the same subset of 50 shapes. The participants were asked to click and drag the shapes into however many separate bins of their choice (max = 10), and to also title each bin with a label of their choice to indicate the category represented by the bin. The participants were

similarly limited to 6 characters in their label to facilitate direct comparisons to the labels produced in the version of the Grouping Game with social interaction. 60 subjects in total were randomized to the nonsocial Grouping Game.

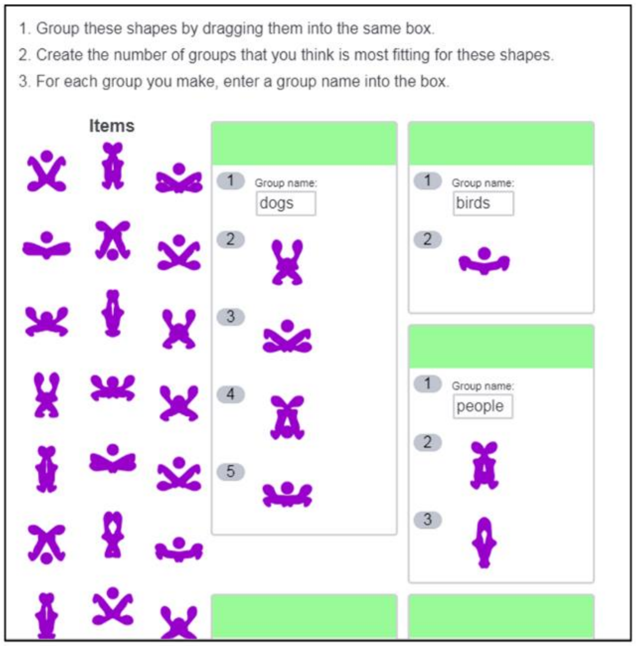


Fig. S6. Screenshot of the individual (nonsocial) version of the Grouping Game.

We observe high levels of divergence among the category systems produced by separate isolated individuals, consistent with the patterns of category divergence observed among dyads (Fig. S7). Less than 2% of labels were shared across independent individuals, despite being shown the exact same stimuli. Fig. S7 shows how 65 unique labels were attempted across a subset of 15 isolated individuals, amounting to over 4 unique labels on average per individual. There was also no consistency in how these isolated individuals partitioned the continuum ($p < .001$, $n = 60$, Kruskal-Wallis H Test). These results are highly consistent with studies indicating substantial variation in the categorization processes of individuals (*9*–*15*).

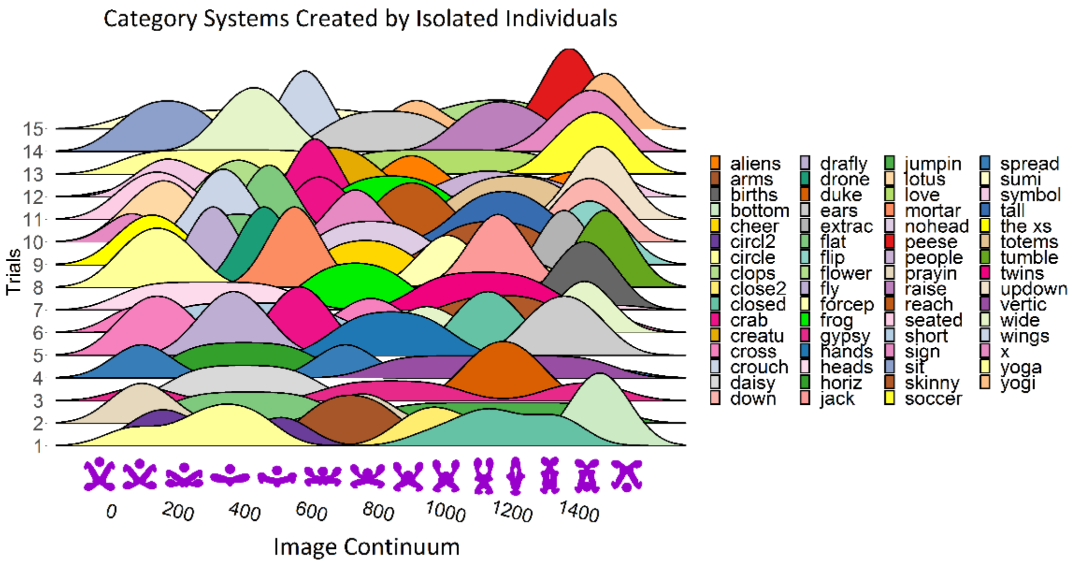


Fig. S7. The category systems that were created by isolated individuals in the $N$=1 version of the Grouping Game. A subset of 15 individuals (trials) are shown from this condition, which contained 60 individuals. Each row displays the category system created by a separate individual. The density distributions display which shapes were placed into the same virtual bin by the particular person corresponding to that row. The colors indicate the label that the individual provided for their bin.

Despite differences in the structure of the nonsocial and social version of the Grouping Game, we find a similar distribution of labels were independently introduced across subjects, suggesting that both versions of the task prompted individuals to initially form similar associations with the stimuli. We find a significant correlation ($p < .001$, $r_s = 0.48$) between the proportion of subjects who independently introduced a label in the solo condition, and the proportion of subjects who independently introduced the same label in the network conditions (aggregated across all social group sizes: 2, 6, 8, 24, and 50). Of particular note, the label "crab" was most popular in both the social and nonsocial version of the Grouping Game, though in no conditions did this label emerge from the majority.

1.8. Robustness to Comparing Category Partition Sets

Here we measure the similarity of how separate vocabularies partitioned the continuum (i.e. how labels grouped together stimuli by identifying boundaries between subsets of shapes in the continuum). To calculate the similarity of partitions imposed by separate category systems, we use Baronchelli et al.'s (2010) measure of centroid overlap (*1*). The centroid of a category is the median image in the image range that each label refers to within the continuum. The distance between centroids is the absolute number of images along the continuum between the centroids of two categories from different trials. For each category $c_i$ in population $X$, our measure calculates the minimum centroid distance between $c_i$ and all categories in population $Y$. It then takes the average across all minimum centroid distances between the two populations as a measure of their overall centroid alignment. To facilitate a more explicit measure of category

convergence across populations, we convert this measure of centroid distance into a measure of centroid overlap by normalizing average centroid distance as $1 - (k_{ij}/m)$, where $k_{ij}$ is the average minimum centroid distance between population $i$ and $j$, and $m$ is the maximum number of images that can separate them (i.e. 1499). The resulting measure indicates the extent to which the partitions imposed by separate category systems overlap along the normalized scale of 0 to 1, where 1 represents perfect alignment.

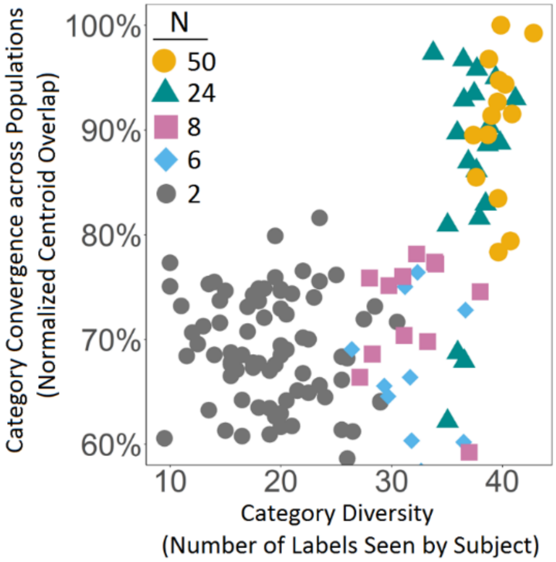


Fig. S8: Measuring category convergence across populations in terms of how the vocabularies of separate groups partitioned the continuum, where the y-axis displays the average overlap between the partitions in a given trial and all other trials of the same group size. The x-axis displays the average number of unique labels encountered over the course of the experiment by each subject within each group. Average partition overlap is quantified using centroid alignment. The centroid of a category is the median image in the range of the continuum to which this category referred. Average partition overlap is measured as $1 - (k_{ij} / m)$, where $k_{ij}$ is the average minimum centroid distance between the categories of population $i$ and $j$, and $m$ is the maximum number of images that can separate two centroids (i.e. 1499).

We report our main results concerning category convergence in terms of the similarity of the vocabularies that emerged among separate groups of the same size. Here we show that the same effect of group size holds if we examine how vocabularies partitioned the continuum, i.e. by marking boundaries between shapes to identify distinct groups. Fig. S8 shows that increasing group size causes a sharp increase in the similarity of continuum partitions that emerged in independent populations of the same size ($p < .001$, $n = 120$, Jonckheere-Terpstra Test). This result illustrates that word choice impacted how populations organized the continuum, and whether they did so in complimentary ways (*16–18*).

1.9. Robustness to the Number of Interactions in a Population

One possible objection is that given more time, dyads would approach the levels of category similarity observed in larger social networks. The concern is that many more interactions happen in parallel in the large networks, such that dyads and smaller social groups are disadvantaged in the overall number of interactions informing their category systems. To test this, we allowed 40 dyads to play for an additional 25 rounds (i.e. for 125 rounds in total). We find that allowing dyads more time to construct their category systems only further entrenched their existing category systems. The average level of category similarity among dyads was not significantly different than dyads that interacted for 100 rounds, in terms of the similarity of the specific labels they adopted ($p = 0.54$, Wilcoxon Rank Sum Test, Two-sided) and how these labels partitioned the continuum ($p = 0.67$, Wilcoxon Rank Sum Test, Two-sided).

1.10. Experimental Design: With Confederates

Subjects were assigned to a fully connected network of 24 participants, where 9 of the 24 participants (i.e. 37%) were confederates trained to use a particular vocabulary for predefined regions of the continuum. The vocabulary of the confederates was chosen to primarily consist of uncommon labels to test whether the confederates could trigger the adoption of uncommon labels for regions of the continuum (Fig. S9). The proportion of confederates was set to 37% to overcome resistance from the 25% of experimental subjects expected to independently introduce common labels (e.g. ‘crab’); prior work (*7*) indicates that resistance of this kind requires new conventions to possess a larger critical mass in order to spread. Fig. S9 displays the vocabulary that the confederates were trained to use.

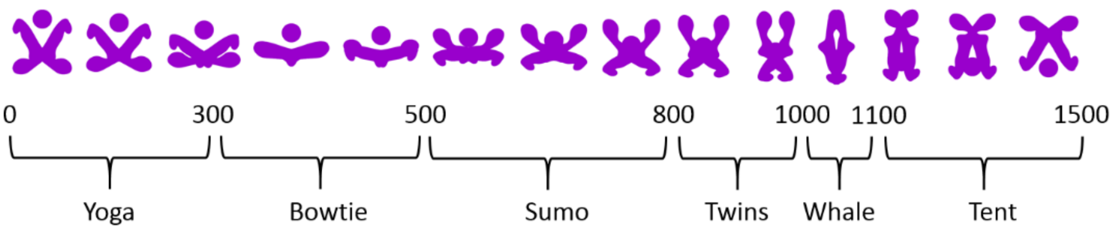


Fig. S9: The vocabulary used by confederates, broken down by continuum region.

Confederates were trained to adhere to their vocabulary in their role as speaker and as hearer. When playing as the speaker on a given round, the confederates identified which region of the continuum the highlighted image corresponded to, and the confederates then provided a predetermined label corresponding to that region. In the role of the hearer, confederates always successfully selected the correct image (i.e. the image referred by the hearer) when the hearer used one of the confederate’s labels. If the nonconfederate speaker was provided with a label that did not belong to the confederates’ vocabulary, the confederate selected images at random.

Consistent with prior work on critical mass dynamics (*7*), confederates behaved in this stubborn manner to directly test whether experimental subjects would adopt alternative labels with sufficient levels of social influence. The confederates interacted with the experimental subjects from the beginning of the experiment until the end, when all experimental subjects had played at least 100 rounds.

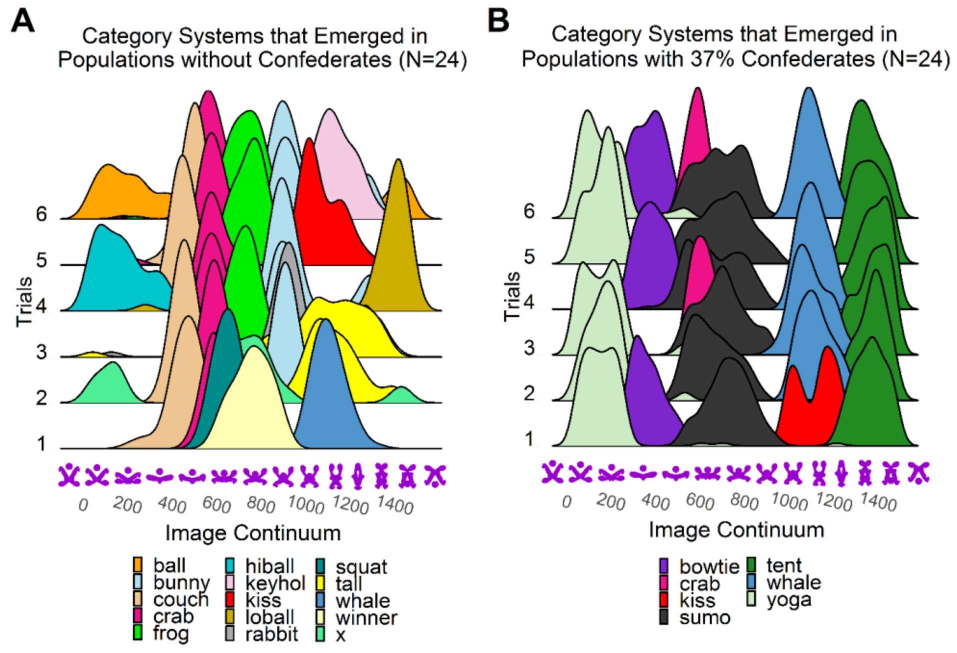


Fig. S10. The category systems that emerged in *N*=24 populations (A) without confederates and (B) with 37% of the network as confederates. Each row displays the category system that emerged in a single trial of the confederate robustness trials, after 100 rounds of interaction. (B) In networks with confederates, each trial consisted of 24 subjects, where 9 were confederates programmed to push infrequent labels, and 15 were experimental subjects unaware of the presence of confederates. In both panels, the density distributions display the frequency of successful coordination for each label, and the region of the image continuum to which each label referred. Each color corresponds to a unique label. The data displayed exclude all interactions among confederates.

1.11. <u>Subject Experience during the Experiment: With Confederates</u>

The subject experience of participants in the experiment with confederates was identical to the subject experience of participants in the experimental trials without confederates. All instructions were identical. The only difference introduced by the experimental trials with confederates is that 9 of the 24 participants in each group were confederates trained to use a particular vocabulary for predefined regions of the continuum (see “Experimental Design: With Confederates”). Fig. S10 displays the category systems that emerged in large populations (*N*=24) with confederates

alongside the category systems that emerged in large populations (*N*=24) without confederates. In every trial, populations adopted the confederates' labels across each region of the continuum, yielding significantly more convergent category systems (58% Jaccard index) than those that emerged in *N*=24 populations without confederates (35% Jaccard index), ($p < .01$, $n = 21$, Wilcoxon rank sum, Two-sided). In the following section ("Supplementary Analyses of Confederate Experiment"), we present statistical analyses suggesting that the confederates influenced subjects' qualitative interpretation of the novel stimuli.

1.12. Supplementary Analyses of Confederate Experiment

We determine whether the confederates influenced subjects' qualitative perceptions of the stimuli by measuring whether the confederates' labels significantly altered the content of subjects' *ad hoc* descriptions of representative images from across the continuum, which subjects provided in an exit survey. As a baseline, we also collected the same exit survey data from 6 networks (*N*=24) in our main experimental trials without confederates. In networks (*N*=24) both with and without confederates, subjects were presented with an exit survey immediately after the coordination task. In this survey, subjects were shown five images selected from the continuum. Next to each image, we displayed the question: "Please describe this image in your own words. What does this image look like to you?" Subjects were then required to input a response of their desired length in a free text-entry window. Each subjects' response was coded to indicate the specific category to which they compared the image. For example, a subject was said to compare an image to a sumo wrestler if their description described the image as a "sumo wrestler", or if it described the image as resembling something similar to a sumo wrestler, such as "man flexing his arms" or as a "wrestler squatting." For each network, we calculated the number of subjects who categorized each image according to the linguistic framing spread by the confederates.

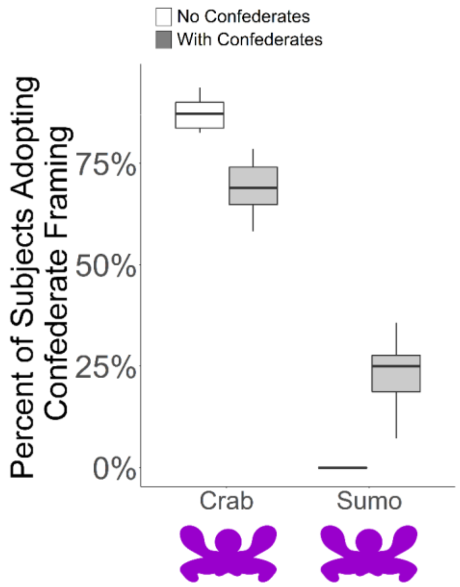


Fig. S11. The effects of confederate influence on subjects' *ad hoc* descriptions of the "crabbiest image" in the continuum (i.e. the image most often labeled as "crab" in the large populations). Horizontal axis compares subjects who framed

the crabbiest image as resembling a "crab" or a "sumo" (where "sumo" was the label advanced by the confederates). Boxplots display the mean percent of subjects across networks in each condition who framed the image shown as resembling a crab or a sumo, differentiated by whether subjects were in networks that either did or did not contain confederates. This measure is averaged within each trial to control for nonindependence. In the trials with confederates, the data display 6 unique social networks of 15 people (9 confederates); and in the condition without confederates, the data display the 6 unique social networks of 24 people for which we collected survey data on subjects' *ad hoc* shape descriptions.

Focusing first on the "crab" region associated with the highest levels of convergence in the main trials (Fig. 2), we find that the vast majority of subjects in the networks without confederates described the 'crabbiest' image from this region as resembling a crab (Fig. S11). By comparison to the main trials, significantly fewer subjects in the networks with confederates described the 'crabbiest image' as a crab, despite its potential cognitive appeal ($p < .001$, $n = 12$, Wilcoxon Rank Sum Test, Two-sided). Strikingly, we find that no subjects in any of the networks without confederates described the "crabbiest" image as resembling a sumo wrestler. By contrast, 23% of subjects on average in each of the networks with confederates described the crabbiest image as resembling a "sumo wrestler", revealing a significant effect of the confederates' influence ($p < .001$, $n = 12$, Wilcoxon Rank Sum Test, Two-sided).

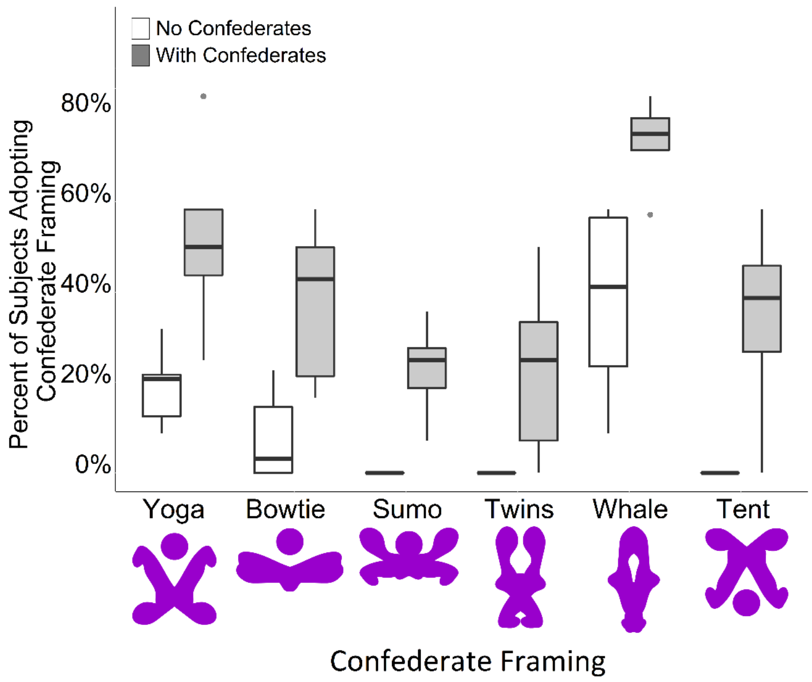

Fig. S12. The effects of confederate influence on subjects' *ad hoc* descriptions of representative images from various regions across the continuum. The horizontal axis displays the image and corresponding category advanced by confederates for this image (and adjacent images) during the experimental task. Boxplots display the percent of subjects across networks in each condition whose *ad hoc* descriptions framed the image shown as resembling the category advanced by the confederates, differentiated by whether subjects were in networks that either did or did not contain confederates. Data points indicate outliers. This measure is averaged within each trial in each condition to control for nonindependence. In the trials with confederates, the data display 6 unique social networks of 15 people (9 confederates); and in the condition without confederates, the data display the 6 unique social networks of 24 people for which we collected survey data on subjects' *ad hoc* shape descriptions.

The confederates' influence was not limited to the 'crab' region of the continuum. Fig. S12 displays the average percentage of subjects in the networks ($N$=24) with and without confederates that described images from across the continuum in terms of the confederates' influence. In each region of the continuum, significantly more subjects in the networks with confederates described the visual stimuli with imagery that resembled the confederates' categories, as compared to subjects in the networks without confederates ($p < .001$, $n = 72$, Wilcoxon Rank Sum Test, Two-sided). Specifically, we find significantly more subjects provided descriptions similar to the confederates' categories in the 0-250 range of the continuum (confederate label "yoga"; $p < .05$, $n = 12$, Wilcoxon Rank Sum Test, Two-sided), in the 250-450 range of the continuum (confederate label "Bowtie"; $p < .05$, $n = 12$, Wilcoxon Rank Sum Test, Two-sided), in the 450-650 range of the continuum (confederates label "Sumo"; $p < .01$, $n = 12$, Wilcoxon Rank Sum Test, Two-sided), in the 800-1000 range of the continuum (confederate label "Twins"; $p < .01$, $n = 12$, Wilcoxon Rank Sum Test, Two-sided), in the 1000-1100 range of the continuum (confederates label "Whale"; $p < .05$, $n = 12$, Wilcoxon Rank Sum Test, Two-sided), and in the 1100-1500 range of the continuum (confederates label "Tent"; $p < .01$, $n = 12$, Wilcoxon Rank Sum Test, Two-sided). Indeed, no subjects in any of the networks without confederates described the images as resembling the categories advanced by the confederates in three regions of the continuum: the 450-650 range (confederate label "Sumo"), the 800-1000 range (confederate label "Twins"), and the 1100-1500 range (confederate label "Tent"). By contrast, in each of these three regions, the confederates achieved significant influence over the final categorical framing that gained adoption (Fig. S12).

Equations

S1. Equation for Fig. 3, derived from our formal model. Eq. $y = x + \mathrm{I}(x^2) + \mathrm{I}(x^3)$

Data Availability: The source data for this study is publicly available at: https://github.com/drguilbe/categories2020;

https://ndg.asc.upenn.edu/uncategorized/network-dynamics-of-category-emergence/

This dataset contains a time series of label production and adoption for each social group in each condition as described in Material and Methods. The data includes for each round: (1) the images in the continuum each player was shown, (2) the image highlighted to be labeled, (3) the label provided by the speaker, (4) and the image clicked on by the hearer (indicating coordination success or failure).

**Supplementary References**


1. Baronchelli, A., Gong, T., Puglisi, A. & Loreto, V. Modeling the emergence of universality in color naming patterns. *Proc. Natl. Acad. Sci. U.S.A.* **107**, 2403–2407 (2010).
2. Baronchelli, A. Sharp transition towards shared vocabularies in multi-agent systems. *Journal of Statistical Mechanics Theory and Experiment* **2006**, P06014–P06014 (2006).
3. Wittgenstein, L. *The blue and brown books*. (HarperCollins, 1965).
4. Puglisi, A., Baronchelli, A. & Loreto, V. Cultural route to the emergence of linguistic categories. *Proc. Natl. Acad. Sci. U.S.A.* **105**, 7936–7940 (2008).
5. Borja, M. C. & Haigh, J. The birthday problem. *Significance* **4**, 124–127 (2007).
6. Munford, A. G. A note on the uniformity assumption in the birthday problem. *The American Statistician* **31**, 119–119 (1977).
7. Centola, D., Becker, J., Brackbill, D. & Baronchelli, A. Experimental evidence for tipping points in social convention. *Science* **360**, 1116–1119 (2018).
8. Winkielman, P., Halberstadt, J., Fazendeiro, T., Catty, S. Prototypes are attractive because they are easy on the mind. *Psychol Sci* **17**, 799–806 (2006).
9. Ranjan, A. & Srinivasan, N. Dissimilarity in creative categorization. *The Journal of Creative Behavior* **44**, 71–83 (2010).
10. Clark, H. H. & Wilkes-Gibbs, D. Referring as a collaborative process. *Cognition* **22**, 1–39 (1986).
11. Spalding, T. & Gregory, M. Effects of background knowledge on category construction. *Journal of Experimental Psychology* **22**, 525–538 (1996).
12. Medin, D. L. & Wattenmaker, W. D., Hampson, S. E. Family resemblance, conceptual cohesiveness, and category construction. *Cognitive Psychology* **19**, 242–279 (1987).
13. Shepard, R. N. & Cermak, G. Perceptual-cognitive explorations of a toroidal set of free-form stimuli. *Cognitive Psychology* **4**, 351–377 (1973).
14. Lindsey, D. T., Brown, A. M., Brainard, D. H. & Apicella, C. L. Hadza color terms Are sparse, diverse, and distributed, and presage the universal color categories found in other world languages. *Iperception* **7**, (2016).
15. Stewart, N. & Chater, N. The Effect of Category Variability in Perceptual Categorization. *Journal of Experimental Psychology: Learning, Memory, and Cognition* **28** (5), 893–907 (2002).
16. Malt, B. C. & Sloman, S. A. Conversation and convention: enduring influences on name choice for common objects. *Mem Cognit* **32**, 1346–1354 (2004).
17. Malt, B. C. & Majid, A., How thought is mapped into words. *Cogn Sci* **4**, 583–597 (2013).
18. Davis, C. P., Morrow, H. M. & Lupyan, G. What does a horgous look like? Nonsense words elicit meaningful drawings. *Cognitive Science* **43**, e12791 (2019).
