# Supplementary material for: Experimental evidence for scale-induced category convergence across populations
Source: Nat Commun. 2021 Jan 12;12:327. doi: 10.1038/s41467-020-20037-y (PMC7804416; doi:10.1038/s41467-020-20037-y)
Supplement: Supplementary file 2 — Reporting Summary [file 41467_2020_20037_MOESM2_ESM.pdf]

nature research

Corresponding author(s): Damon Centola

Last updated by author(s): Sep 16, 2020

Reporting Summary

Nature Research wishes to improve the reproducibility of the work that we publish. This form provides structure for consistency and transparency in reporting. For further information on Nature Research policies, see our Editorial Policies and the Editorial Policy Checklist.

Statistics

For all statistical analyses, confirm that the following items are present in the figure legend, table legend, main text, or Methods section.

n/a	Confirmed	
☐	☒	The exact sample size (n) for each experimental group/condition, given as a discrete number and unit of measurement
☐	☒	A statement on whether measurements were taken from distinct samples or whether the same sample was measured repeatedly
☐	☒	The statistical test(s) used AND whether they are one- or two-sided<br>*Only common tests should be described solely by name; describe more complex techniques in the Methods section.*
☐	☒	A description of all covariates tested
☐	☒	A description of any assumptions or corrections, such as tests of normality and adjustment for multiple comparisons
☐	☒	A full description of the statistical parameters including central tendency (e.g. means) or other basic estimates (e.g. regression coefficient) AND variation (e.g. standard deviation) or associated estimates of uncertainty (e.g. confidence intervals)
☐	☒	For null hypothesis testing, the test statistic (e.g. F, t, r) with confidence intervals, effect sizes, degrees of freedom and P value noted<br>*Give P values as exact values whenever suitable.*
☒	☐	For Bayesian analysis, information on the choice of priors and Markov chain Monte Carlo settings
☒	☐	For hierarchical and complex designs, identification of the appropriate level for tests and full reporting of outcomes
☒	☐	Estimates of effect sizes (e.g. Cohen's d, Pearson's r), indicating how they were calculated

Our web collection on statistics for biologists contains articles on many of the points above.

Software and code

Policy information about availability of computer code

Data collection	The data were collected using a proprietary web platform called "The Grouping Game."
Data analysis	The code used in analyzing the data in this study were custom built in R. All code relevant for the reproduction of our statistical analyses is publicly available at the following github: https://github.com/drguilbe/categories2020

For manuscripts utilizing custom algorithms or software that are central to the research but not yet described in published literature, software must be made available to editors and reviewers. We strongly encourage code deposition in a community repository (e.g. GitHub). See the Nature Research guidelines for submitting code & software for further information.

Data

Policy information about availability of data

All manuscripts must include a data availability statement. This statement should provide the following information, where applicable:

- Accession codes, unique identifiers, or web links for publicly available datasets
- A list of figures that have associated raw data
- A description of any restrictions on data availability

Data Availability: The data for this study are publicly available at the following github: https://github.com/drguilbe/categories2020

Field-specific reporting

Please select the one below that is the best fit for your research. If you are not sure, read the appropriate sections before making your selection.

☐ Life sciences ☒ Behavioural & social sciences ☐ Ecological, evolutionary & environmental sciences

For a reference copy of the document with all sections, see nature.com/documents/nr-reporting-summary-flat.pdf

Behavioural & social sciences study design

All studies must disclose on these points even when the disclosure is negative.

Study description

This is a quantitative, experimental study of how populations of different sizes construct category systems for a continuum of novel and arbitrary stimuli.

Research sample

The research sample consists of online workers from the popular crowdsourcing platform Amazon Mechanical Turk. All workers were based in the U.S., and there were no differences in the distribution of demographic traits across conditions. Specifically, there were no differences in the distribution of demographic traits across conditions, in terms of gender ($p = 0.56$), ethnicity ($p = 0.42$), and age ($p = 0.67$), (Kruskal-Wallis H Test). 52% of subjects identified as 'Female'; 47% of subjects identified as 'Male'; 1% of subjects identified as 'Non-binary'. 7% of subjects were 18-24 years old; 30% were 25-34 years old; 34% were 35-44 years old; 14% were 45-54 years old; 8% were 55-64 years old; 7% were above 65 years old. 47% of our sample identified as Non-white. The Mturk sample may not be nationally representative, but methodological research has found that Mturk subjects provide high quality data for modeling human behavior, as compared to traditional survey methods (see reference 1 & 2 below).

1. A Berinsky, G Huber, G Lenz. Evaluating Online Labor Markets for Experimental Research: Amazon.Com's Mechanical Turk. Political Analysis 20(3), 351–68 (2012).

2. L Litman, J Robinson, T Abberbock. TurkPrime.Com: A Versatile Crowdsourcing Data Acquisition Platform for the Behavioral Sciences. Behavior Research Methods 49(2), 433–42 (2017).

Sampling strategy

Power tests were calculated for the Wilcoxon Signed Rank test to determine sufficient sample sizes.

Data collection

Upon arriving to the study, participants viewed instructions on how to play the game. The Grouping Game infrastructure then automatically and randomly assigned participants to an experimental condition. For each experimental trial, subjects were randomly assigned to a fully connected network (i.e. homogeneously mixing population) of either 2, 6, 8, 24, or 50 people. Each condition in each trial consisted of unique individuals. We collected 80 independent dyads ($N=2$) and 15 independent social groups for every other group size ($N=6,8,24,50$). The lead author, Douglas Guilbeault, was present for the data collection and operated the instruments used to collect the data. The system automatically assigned subjects to condition in a way that all researchers were blind to as part of the data collection process.

Timing

Data was collected between September 2018 and February 2020.

Data exclusions

10 trials were removed from the dyad condition because one of the members of the dyad dropped out during the experiment, such that the remaining partner had no one to play with. These null trials were removed. Otherwise, no data was excluded from the analysis.

Non-participation

Less than 3% of subjects exhibited attrition during the task, and this attrition rate was randomly distributed across conditions. Since all analyses and comparisons are at the group-level, these attrition rates do not impact the statistical power of our between condition comparisons.

Randomization

All participants were automatically randomized to condition, regardless of their demographic traits.

Reporting for specific materials, systems and methods

We require information from authors about some types of materials, experimental systems and methods used in many studies. Here, indicate whether each material, system or method listed is relevant to your study. If you are not sure if a list item applies to your research, read the appropriate section before selecting a response.

Materials & experimental systems

n/a	Involved in the study
☒	☐ Antibodies
☒	☐ Eukaryotic cell lines
☒	☐ Palaeontology and archaeology
☒	☐ Animals and other organisms
☐	☒ Human research participants
☒	☐ Clinical data
☒	☐ Dual use research of concern

Methods

n/a	Involved in the study
☒	☐ ChIP-seq
☒	☐ Flow cytometry
☒	☐ MRI-based neuroimaging

Human research participants

Policy information about studies involving human research participants

Population characteristics	There were no differences in the distribution of demographic traits across conditions, in terms of gender (p = 0.56), ethnicity (p = 0.42), and age (p = 0.67), (Kruskal-Wallis H Test). 52% of subjects identified as 'Female'; 47% of subjects identified as 'Male'; 1% of subjects identified as 'Non-binary'. 7% of subjects were 18-24 years old; 30% were 25-34 years old; 34% were 35-44 years old; 14% were 45-54 years old; 8% were 55-64 years old; 7% were above 65 years old. 47% of our sample identified as Non-white.
Recruitment	Subjects were recruited from Amazon's Mechanical Turk. The only requirement imposed on recruitment is that subjects had to be based in the U.S. Otherwise, subjects were recruited regardless of their demographics. Our sample required subjects to be available to play an online game for over 1 hour continuously, which may have excluded certain subjects or may have supported self-selection based on interest in the hit. Yet, the following two facts suggest that our results are robust to any variation in Mturk's demographic: (1) all subjects were asked to categorize an ambiguous continuum of stimuli that was novel to everyone, regardless of their background; and (2) we observed a rich variety of categories proposed in the experiment, suggesting that any contributions of self-selection are unable to account for the effects of larger populations on promoting replicable evolutionary trajectories toward similar category systems.
Ethics oversight	This study was approved by the IRB board at the University of Pennsylvania.

Note that full information on the approval of the study protocol must also be provided in the manuscript.
